# Developmental Dysplasia of the Hip with Concurrent Legg-Calvé-Perthes Disease in the Contralateral Hip

**DOI:** 10.7759/cureus.6494

**Published:** 2019-12-28

**Authors:** Majed Al Osaimi, Ahmed Sonbul, Ahmed Ibrahim

**Affiliations:** 1 Surgery, King Abdulaziz Medical City / Ministry of National Guard - Health Affairs, Jeddah, SAU; 2 Orthopaedic Surgery, King Abdulaziz Medical City / Ministry of National Guard - Health Affairs / King Saud Bin Abdulaziz University / King Abdullah International Medical Research Center, Jeddah, SAU; 3 Orthopaedics, King Saud Bin Abdulaziz University for Health Sciences / College of Medicine, Jeddah, SAU

**Keywords:** ddh, lcpd, avn

## Abstract

Developmental dysplasia of the hip (DDH) is a common hip disorder in pediatrics; about one in 100 newborns has it due to dysplasia and one to two per 1000 have it due to dislocation. Some factors are responsible for the disease, and breech presentation has been identified as a major risk factor. It might be associated with other conditions too. Patients with Legg-Calvé-Perthes disease present with painless limping gait with idiopathic etiology; it is unilateral in most of the cases. This paper reported a rare scenario of DDH associated with concurrent contralateral Legg-Calvé-Perthes disease.

A 5-year-old Saudi male patient, known case of developmental dysplasia of the right hip, which was managed operatively at a different hospital, presented in our outpatients clinic for right hip dysplasia and was found incidentally as having a limping gait due to left hip limited range of motion, following clinical assessment, pelvic radiographs demonstrated presence of subluxation at the right hip in addition to sclerosis, and irregularity of the left femoral head epiphysis. Right pelvic Dega osteotomy, femoral derotation osteotomy, varus osteotomy, and left hip arthrogram was examined under anesthesia with the positioning of the left hip at 45 degrees of abduction and 30 degrees of flexion to achieve the best coverage. Hip spica application was performed to correct DDH of the right hip. Repeated radiography at the subsequent visits showed better coverage of the femoral head on the right side. During a routine follow-up, there were also some osteonecrotic changes of the left femoral head that confirmed the diagnosis of left Legg-Calvé-Perthes disease.

## Introduction

Developmental dysplasia of the hip (DDH), previously known as congenital dislocation of the hip (CDH), is an umbrella term that includes a wide spectrum of disorders in which the femoral head has an abnormal relationship to the acetabulum [1]. These conditions might present with acetabular dysplasia, subluxation, dislocation, or teratologic dislocation of hip (which is frequently seen with arthrogryposis, myelomeningocele, Larsen syndrome, and Ehlers-Danlos syndrome), and adolescent dysplasia [1].

There are many reports of DDH associated with other conditions, such as congenital muscular torticollis, metatarsus adductus, and congenital knee dislocation [1-2]. We report the first case of DDH with concurrent Legg-Calvé-Perthes disease (LCPD) in the contralateral hip in Saudi Arabia, after approval of the institutional review board (IRB) of King Abdullah International Medical Research Center (KAIMRC) at Ministry of National Guard Health Affairs (MNGHA), Jeddah, Kingdom of Saudi Arabia.

## Case presentation

A five-year-old Saudi boy, known case of developmental dysplasia of the right hip, otherwise healthy, presented at the outpatient clinic of King Khalid Hospital, King Abdulaziz Medical City, Jeddah, because of an abnormal gait. He had undergone treatment earlier at another hospital, first with closed reduction and then with open reduction.

**Figure 1:**
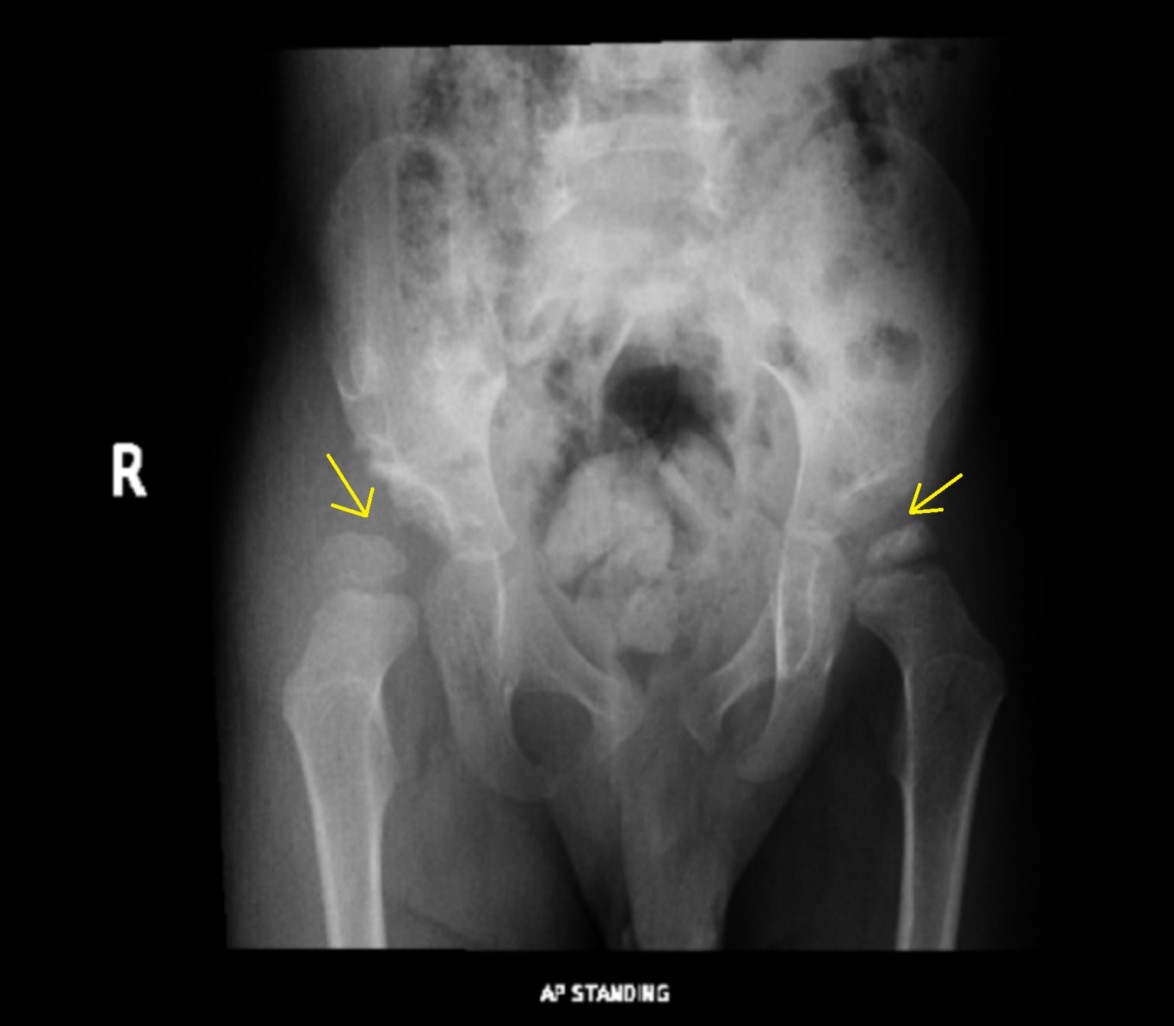
AP view: Shows right hip subluxation and left femoral head epiphysis sclerosis AP: anteroposterior

**Figure 2:**
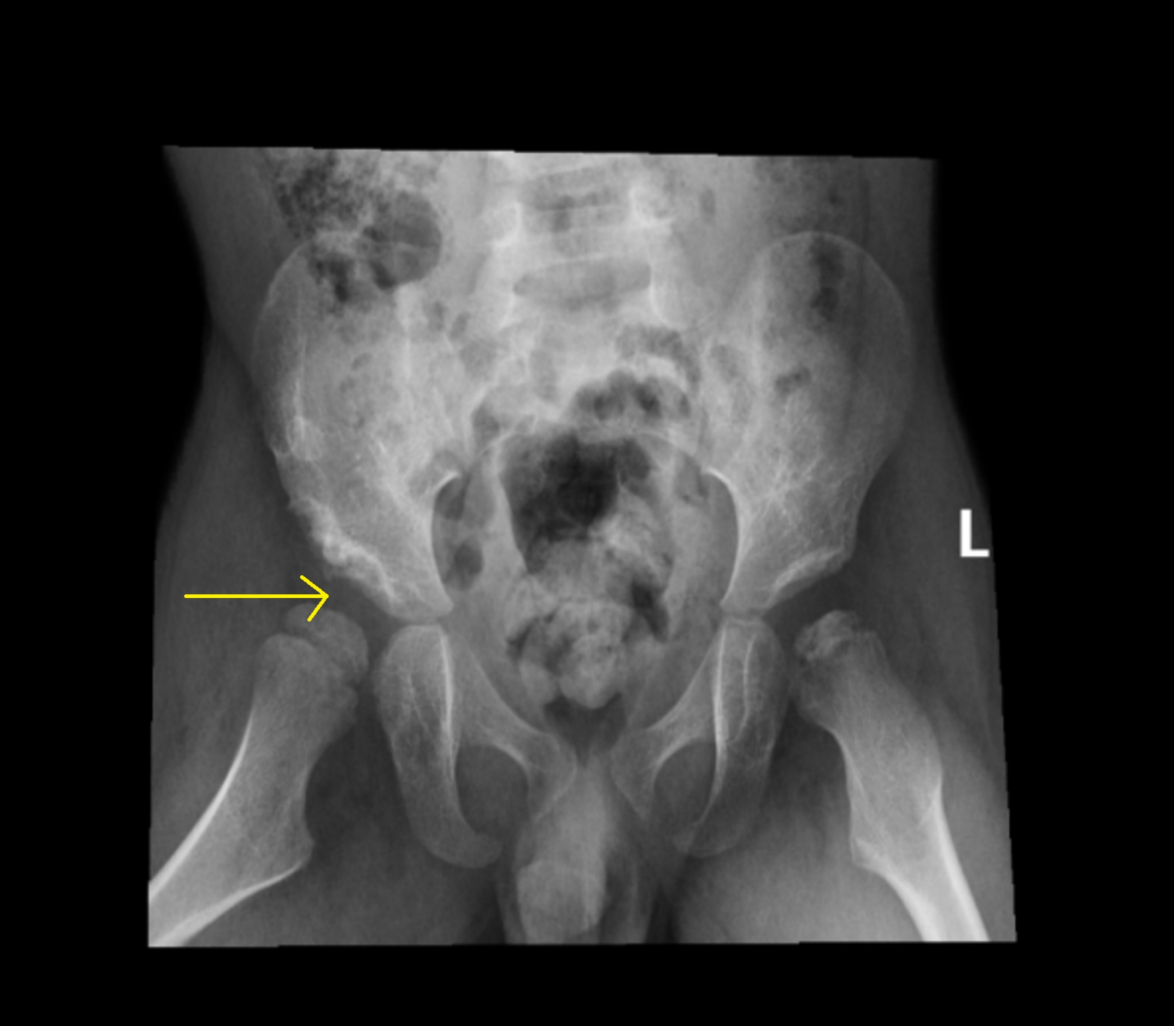
Frog-lateral view: Shows a dysplastic right acetabulum with a superolateral subluxation femoral head

Upon examination, the child was found to have a limping gait, decreased range of motion (pronounced in abduction) of the left hip, and a positive Galeazzi sign. Anteroposterior radiograph of the pelvis and the frog-lateral view showed right DDH with persistent right hip subluxation (Figure 1 and Figure 2).

**Figure 3:**
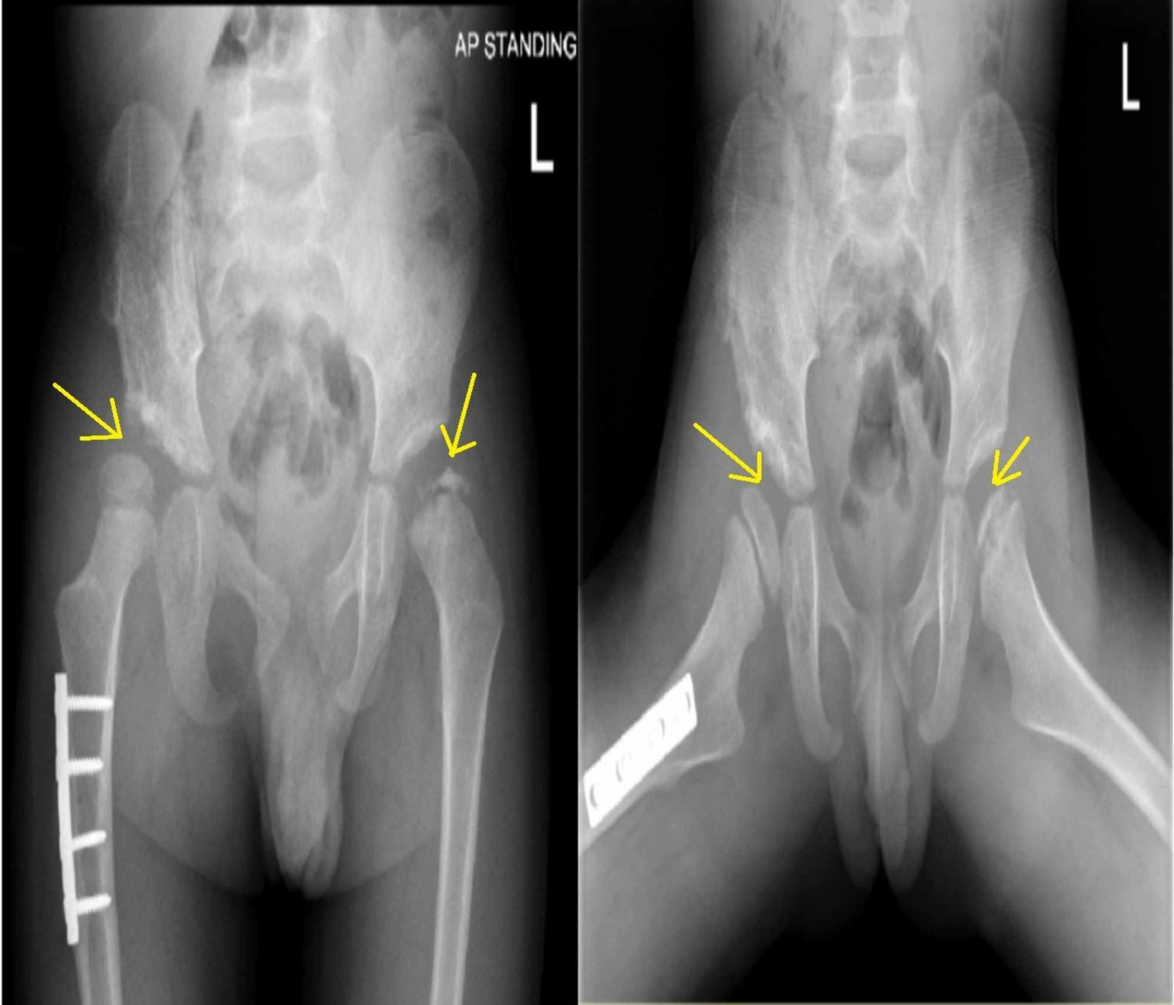
AP and frog-lateral views: Right hip shows better coverage and femoral head with mild subluxation and healed osteotomy while left hip shows sclerosis, irregularity, and fragmentation of the left femoral head epiphysis AP: anteroposterior

**Figure 4:**
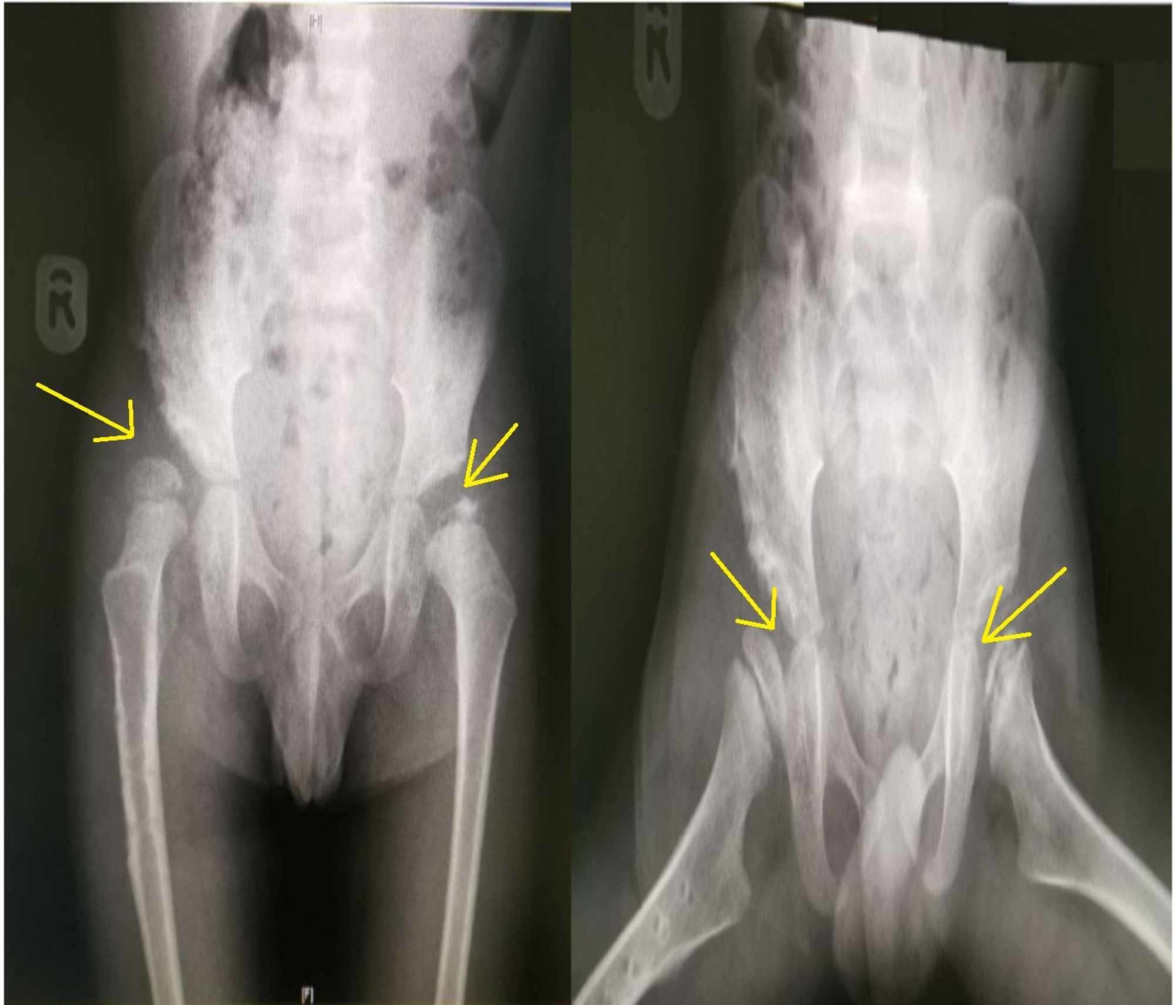
AP and frog-lateral views: Post removal of plate and screws of the right hip with better coverage; left hip in the fragmentation stage (Legg-Calvé-Perthes disease) AP: anteroposterior

The left hip was also abnormal, with sclerosis and irregularity of the left femoral head epiphysis (Figure 1). The need for surgical intervention was explained to the parents, and the boy was admitted for elective surgery. Bilateral hip arthrogram, right pelvic Dega osteotomy, right femoral varus derotation osteotomy, and left hip examination under anesthesia, with positioning of the left hip at 45 degrees of abduction and 30 degrees of flexion to achieve the best coverage, and hip spica application were performed. Surgery was uneventful, and the child was discharged in good condition. He was followed up in the outpatient department. At each visit, the radiograph of the pelvis was repeated. The right hip showed a progressive improvement, with an acceptable acetabular index and mild sublaxation of the femoral head, and good remodeling (Figure 3 and Figure 4). The left hip showed avascular necrosis (AVN) of the head of the femur and mild subluxation, confirming the diagnosis of Legg-Calvé-Perthes disease in the fragmentation stage (Figure 3).

## Discussion

DDH is one of the most common hip disorders seen in children; about one in 100 newborns have hip dysplasia and one to two per 1000 have hip dislocation [2-5]. Many genetic and environmental factors are responsible for DDH [1-2]. The primary cause is the instability of the joint due to ligamentous laxity, which may be due to genetic or mechanical factors. Breech presentation has been identified as a major risk factor; others include family history, female gender, birth order (with a higher risk in the firstborn), and oligohydramnios. Early diagnosis and treatment of DDH can ensure a better functional outcome and prevent degenerative joint disease [1]. Clinical screening of newborns for DDH is now universal. Ultrasonography is also used for the diagnosis of DDH in infants less than three months old. In addition, ultrasonography is used as a screening tool for neonates in some developed countries, especially for infants with risk factors [6-7]. Pelvic radiography is used after the age of five months to confirm the diagnosis.

The primary treatment of DDH is with the Pavlik harness application and has a success rate of approximately 90% in infants less than three months of age [1,8]. When Pavlik harness treatment fails, closed or open reduction is undertaken, depending on the severity of the condition and age of the child [1,9-10]. Avascular necrosis of the femoral head is a serious complication following treatment and is mainly associated with improper hip spica application or iatrogenic by injuring the blood supply of the femoral head such as by utilizing the medial approach in open reduction. It can lead to acetabular dysplasia, joint incongruity, limb length discrepancy, and early osteoarthritis as a long-term consequence. In our patient, avascular necrosis was seen in the non-operated normal hip, which was an unusual presentation, and it was discovered that the patient has Legg-Calvé-Perthes disease of the contralateral healthy hip [9-10].

Legg-Calvé-Perthes disease is an idiopathic disorder [11-12]. It is unilateral in 89% of cases [11]. Symptoms are mild and nonspecific in early stages; moreover, young children may not be able to describe symptoms [9,12]. The clinical signs depend on the time of presentation and the age of the patient. Limping gait and limited hip range of motion are due to the shortening of the ipsilateral gluteal musculature, which leads to hip abductor deficiency [11-12]. Without a functional abductor musculature, the pelvis tilts to the contralateral side during the stance phase, and the patient leans toward the affected side in compensation (Trendelenburg sign) [11- 12]. The range of motion of the hip joint varies with the stage of the disease.

The goal of treatment in Legg-Calvé-Perthes disease is to restore range of motion and maintain adequate coverage of the femoral head to prevent deformation. However, the treatment depends on the age and stage of the presentation, the extent of involvement, and the range of motion of and the presence of extrusion of the femoral head [13-14]. For young children less than five years of age, non-operative management, such as non-weight bearing, using a walking aid, an abduction brace, or Petrie casting, is used. This was found to have good outcomes, especially in patients of a younger age [13,15]. Surgical containment was used to maintain normal femoral head sphericity during revascularization. Proximal femoral varus osteotomy was performed with varus open wedge osteotomy, trochanteric epiphysiodesis, and external rotation, no external immobilization is needed following the surgery while the fixation is kept up until complete healing and remodeling occur [15]. The use of a combined salter and proximal femoral varus osteotomies is effective for severe disease in older patients, in children more than eight years of age with severe subluxation, and in those children where either single femur or innominate osteotomy is insufficient to provide adequate containment [15]. The normal range of motion of the hip joint is a prerequisite for surgical containment. An abduction cast for six weeks is preferred before considering surgical containment in children who fail to regain normal range of motion after a few days of traction [14-15].

## Conclusions

This is the first report from Saudi Arabia of DDH of one hip with concurrent contralateral Legg-Calvé-Perthes disease. It is essential to report such cases, as these diseases have not been reported in the literature to occur simultaneously. It is recommended to report such cases to have an incidence figure and to correlate and investigate risk factors. This paper discussed each disease distinctively and correlated the risk factors, pathogenesis, and patient outcomes.
